# Shortages of benzathine benzylpenicillin G in Australia highlight the need for new sovereign manufacturing capability

**DOI:** 10.5694/mja2.52590

**Published:** 2025-01-20

**Authors:** Rosemary Wyber, Glenn Pearson, Laurens Manning

**Affiliations:** ^1^ The Kids Research Institute Australia Perth WA; ^2^ Yardhura Walani Australian National University Canberra ACT; ^3^ University of Western Australia Perth WA

**Keywords:** Rheumatic heart disease, Anti‐infective agents

Benzathine benzylpenicillin G (BPG) is the most effective treatment for syphilis and prevention of rheumatic heart disease (RHD), both of which disproportionately affect Aboriginal and Torres Strait Islander people. The ongoing syphilis epidemic in Australia^1^ highlights the importance of a reliable supply of high quality BPG in achieving Australia's commitments to ending RHD and preventing new cases of congenital syphilis.^2^

BPG is a long‐acting penicillin. After intramuscular injection, the BPG crystals slowly release penicillin into the bloodstream, providing sustained concentrations, which prevent the recurrent streptococcal infections that lead to RHD. These sustained concentrations can also treat established syphilis infections. The BPG manufacturing process and mode of delivery has remained largely unchanged since the 1950s. Australia, along with other high income countries, has imported Bicillin‐LA (Pfizer), the only BPG preparation with Therapeutic Goods Administration (TGA) approval since the 1990s.^3^ Most low and middle income countries use a lyophilised powder formulation, which, unlike a prefilled syringe, requires mixing with a diluent at administration.

At a global level, fragmented BPG manufacturing, supply and procurement has led to recurrent global shortages, both of Bicillin‐LA and powdered formulations. These shortages have led to an increased incidence of syphilis cases.^1^ There are four manufacturers of the active pharmaceutical ingredient (API); three in China and one in Austria. The Chinese companies produce 95% of the global supply but only the Austrian company produces API under certified Good Manufacturing Practice conditions. Due to low profitability, production of powdered BPG is only triggered by large minimum orders. Large procurement agencies are unable to smooth out supply constraints because of a lack of confidence in manufacturing quality.^4^

## Responses to BPG shortages

Due to a lack of sovereign manufacturing capacity, Australia is vulnerable to shortages of World Health Organization‐listed essential medicines, such as BPG. In the early 2000s, there were short supply disruptions of Bicillin‐LA, followed by an extended stockout from 2006 to 2008. During this stockout, a Section 19A exemption to the *Therapeutic Goods Act 1989* (Cwlth) was secured from the TGA to import a powdered formulation (Pan Benz; Panpharma, France) and there were haphazard efforts to support clinician awareness about how to use the preparation.^3^ Shorter supply disruptions over subsequent decades have also affected Australia. In late 2023, the TGA was notified by Pfizer of an expected stockout lasting into mid‐2024.^5^

Australia's response to the predicted stockout was swift. A Section 19A approval for a powdered product (Brancaster Pharma, UK) was secured in late 2023 and listed on the Pharmaceutical Benefits Scheme from 1 January 2024 and another powdered product in early 2024 (Extencilline, France).^4^, ^5^ During this time, a wide range of organisations contributed to the preparations needed for introduction of the alternative product, including understanding the stockout, forecasting the duration and providing guidance on stock management. The National Aboriginal Community Controlled Health Organisation (NACCHO) was instrumental in disseminating key information to end users about the change in formulation and delivery methods. This included webinars and newsletter updates, alongside robust resources from the Australian Commission on Quality and Safety in Health Care and other organisations.^6^, ^7^

Despite these efforts, shortages of a TGA‐approved prefilled BPG preparation have had unacceptable consequences for people living with RHD and syphilis. The recently approved powdered product is reconstituted in an injection volume of 4.5 mL, nearly twice the volume of the existing prefilled BPG product.^6^ Injection volume is a major determinant of pain level on administration. Even at 2.3 mL, most people describe receiving Bicillin‐LA injections as painful.^8^ To prevent RHD, Aboriginal and Torres Strait Islander people at risk of RHD receive these injections every 28 days for a minimum of five years, and any increase in pain is a further unacceptable consequence. There remain difficulties in accessing the recently approved powdered BPG preparation. This has led to the increase in prescriptions of oral penicillin preparations, which are less effective than BPG.^9^

## Preventing future shortages

Australia cannot meet Closing the Gap targets or other targets for syphilis and RHD control without a reliable supply of a high quality BPG preparation. The Aboriginal Community Controlled Health Organisation sector and partners have demonstrated considerable capacity to respond to this health emergency but no one can procure product when no product exists. Stockouts and supply disruptions have been a feature of this drug for nearly two decades and ongoing international instability of supply is anticipated.^10^ In addition to domestic effects, shortages are affecting or are anticipated to affect our neighbours in New Zealand (Aotearoa) and across the Asia–Pacific region. Plans for the prevention and management of shortages of BPG and other medicines are needed.

Responses to a recent TGA inquiry suggest that clearer governance structures and role definition of stakeholders are key priorities needed to improve Australia's response to medicine shortages.^11^ The role of NACCHO and community‐controlled organisations should be recognised as part of this, especially for medicines with particular relevance for Aboriginal and Torres Strait Islander people. Recent examples include shortages of permethrin cream (for treatment of scabies), azithromycin (for treatment of wet cough and trachoma) and glucagon‐like peptide‐1 receptor agonists for management of type 2 diabetes. Mechanisms that address the disproportionate impact of medicine supply issues for Aboriginal and Torres Strait Islander people are consistent with agreed national priorities for Closing the Gap.

Additionally, as part of its commitment to Aboriginal and Torres Strait Islander health and regional health security, Australia should consider specific proactive responses to BPG shortages. This should include exploring sovereign manufacturing capacity to produce locally formulated BPG from quality assured, internationally produced API, such as newly increased supply by Sandoz in Austria.^12^ Local formulation of this API could build on Australia's history as a penicillin manufacturer^13^ and amplify impact of new investments in pharmaceutical facilities in Western Australia.^14^ Although Australia's pharmaceutical sector remains small by global standards, strategic investment in domestically important products is an agreed, resourced national priority.^15^ Onshore pharmaceutical manufacturing is a major capital expense — and cannot provide for all of Australia's medication needs — but does have a role for specific products, as recently illustrated by leadership in mRNA vaccine manufacture.^16^ Sovereign manufacturing of BPG should form part of a comprehensive approach to securing reliable supply of pharmaceuticals for Australia, including support for global supply networks and collaboration between governments.

Further research investment into promising new approaches to manufacture and delivery, led by Australian researchers should also be accelerated. This includes recent data supporting subcutaneous (rather than intramuscular) delivery, which is less painful and lasts longer, resulting in fewer injections.^17^, ^18^ With this new knowledge and an investment strategy to develop and support local manufacture, Australia could become a global leader in efforts to control these devastating infectious diseases.

## Open access

Open access publishing facilitated by Australian National University, as part of the Wiley – Australian National University agreement via the Council of Australian University Librarians.

## Competing interests

No relevant disclosures.

## Provenance

Not commissioned; externally peer reviewed.
